# Distribution patterns of molecular markers of antimalarial drug resistance in *Plasmodium falciparum* isolates on the Thai-Myanmar border during the periods of 1993–1998 and 2002–2008

**DOI:** 10.1186/s12864-023-09814-3

**Published:** 2024-03-11

**Authors:** Phunuch Muhamad, Papichaya Phompradit, Wanna Chaijaroenkul, Kesara Na-Bangchang

**Affiliations:** 1 Drug Discovery and Development Center, Office of Advanced Science and Technology, Thammasat University, Pathumthani, 12120 Thailand; 2 Chulabhorn International College of Medicine, Thammasat University, Pathumthani, 12120 Thailand; 3 Center of Excellence in Pharmacology and Molecular Biology of Malaria and Cholangiocarcinoma, Chulabhorn International College of Medicine, Thammasat University, Pathumthani, 12120 Thailand; 4 Graduate Program in Bioclinical Sciences, Chulabhorn International College of Medicine, Thammasat University, Pathumthani, 12120 Thailand

**Keywords:** *Plasmodium falciparum*, *Plasmodium falciparum* chloroquine resistance transporter (*pfcrt*), *Plasmodium falciparum* multi-drug resistance 1 (*pfmdr1*), *Plasmodium falciparum* kelch 13-propeller (*pfk13*)

## Abstract

**Background:**

Polymorphisms of *Plasmodium falciparum* chloroquine resistance transporter (*pfcrt*), *Plasmodium falciparum* multi-drug resistance 1 (*pfmdr1*) and *Plasmodium falciparum* kelch 13-propeller (*pfk13*) genes are accepted as valid molecular markers of quinoline antimalarials and artemisinins. This study investigated the distribution patterns of these genes in *P. falciparum* isolates from the areas along the Thai-Myanmar border during the two different periods of antimalarial usage in Thailand.

**Results:**

Polymerase chain reaction-restriction fragment length polymorphism (PCR-RFLP) were used to detect *pfcrt* mutations at codons 76, 220, 271, 326, 356, and 371 as well as *pfmdr1* mutation at codon 86. The prevalence of *pfcrt* mutations was markedly high (96.4–99.7%) in samples collected during both periods. The proportions of mutant genotypes (number of mutant/total isolate) at codons 76, 220, 271, 326, 356 and 371 in the isolates collected during 1993–1998 (period 1) compared with 2002–2008 (period 2) were 97.9% (137/140) vs. 97.1% (401/413), 97.9% (140/143) vs. 98.8% (171/173), 97.2% (139/143) vs. 97.1% (333/343), 98.6% (140/142) vs. 99.7% (385/386), 96.4% (134/139) vs. 98.2% (378/385) and 97.8% (136/139) vs. 98.9% (375/379), respectively. Most isolates carried *pfmdr1* wild-type at codon 86, with a significant difference in proportions genotypes (number of wild type/total sample) in samples collected during period 1 [92.9% (130/140)] compared with period 2 [96.9% (379/391)]. Investigation of *pfmdr1* copy number was performed by real-time PCR. The proportions of isolates carried 1, 2, 3 and 4 or more than 4 copies of *pfmdr1* (number of isolates carried correspondent copy number/total isolate) were significantly different between the two sample collecting periods (65.7% (90/137) vs. 87.8% (390/444), 18.2% (25/137) vs. 6.3%(28/444), 5.1% (7/137) vs. 1.4% (6/444) and 11.0% (15/137) vs. 4.5% (20/444), for period 1 vs. period 2, respectively). No *pfk13* mutation was detected by nested PCR and nucleotide sequencing in all samples with successful analysis (n = 68).

**Conclusions:**

The persistence of *pfcrt* mutations and *pfmdr1* wild-types at codon 86, along with gene amplification in *P. falciparum*, contributes to the continued resistance of chloroquine and mefloquine in *P. falciparum* isolates in the study area. Regular surveillance of antimalarial drug resistance in *P. falciparum*, incorporating relevant molecular markers and treatment efficacy assessments, should be conducted.

## Background

Multidrug-resistant *Plasmodium falciparum* remains a significant public health problem along the international borders of Thailand [1]. Several antimalarial drugs have been introduced for clinical uses to overcome the problem. Chloroquine was the first antimalarial drug that was used as the first-line treatment for acute uncomplicated *P. falciparum* until the report of chloroquine resistance along the Thai-Cambodian border in the late 1960s. The combination of sulfadoxine-pyrimethamine (S/P) replaced chloroquine in the early 1970s; however, parasites developed resistance to this combination within a decade. Mefloquine, combined with S/P (MSP), was introduced as first-line treatment in 1985. The parasites rapidly developed resistance to MSP within five years of its clinical use, and mefloquine monotherapy was recommended to replace MSP in 1991 [2, 3]. Due to high failure rates of mefloquine therapy, the artemisinin-based combination therapy (ACT)-- artesunate-mefloquine was introduced as the first-line treatment for multi-drug resistant *P. falciparum* malaria in 1995 [4], and later on, dihydroartemisinin-piperaquine combination in 2010 [5]. Nevertheless, a high treatment failure rate (49.4%) was reported in Srisaket Province on the Thai-Cambodian border in 2019, and the treatment was replaced by artesunate-pyronaridine at the provincial level [6].

Adaption of the parasite’s biology at the molecular level could significantly affect the sensitivity of *P. falciparum* to antimalarial drugs. Studies on molecular markers of antimalarial resistance are essential for monitoring the emergence, spread, and evolution of antimalarial drug resistance and, thus, treatment policy. The mutations of *P. falciparum* chloroquine resistance transporter (*pfcrt*) at codons K76T, A220S, Q271E, N326S, I356T and R371I and *P. falciparum* multi-drug resistance 1 (*pfmdr1*) at codon N86Y have been reported to link with chloroquine resistance in *P. falciparum* [7, 8]. The presence of *pfcrt* and *pfmdr1* mutations can modify chloroquine sensitivity to the high resistance level [9, 10]. On the other hand, the *pfmdr1* mutations at codons 86, 1034 and 1042 improve the sensitivity of the parasites to mefloquine, halofantrine, and lumefantrine [11]. *Pfmdr1* gene amplification is a solid molecular marker of mefloquine resistance and has been proposed to predict treatment failure of ACTs [12, 13]. For artemisinin resistance, strong correlations have been reported between *pfkelch 13*-propeller (*pfk13*) mutations and in vitro parasite survival rates and *in vivo* parasite clearance rates [14]. The mutation of *pfk13* at codon C580Y or F446I is associated with delayed parasite clearance time and artemisinin resistance [14, 15]. In addition, *pfmdr1* gene amplification together with *pfk13* gene mutation well predict ACTs treatment response [16].

The present study investigated the distribution patterns of the molecular markers of antimalarial drug resistance, *i.e., pfcrt*, *pfmdr1* and *pfk13* in *P. falciparum* isolates collected during 1993–1998 and 2002–2008. The selected periods were based on clinical uses of antimalarial drug regimens in Thailand. The samples collected during period 1 (1993–1998) represent *P. falciparum* isolates suspended from chloroquine exposure for about 30 years, while they were exposed to mefloquine mono- or combination therapy. The samples collected during period 2 (2002–2008) represent the parasite population after about 40 years of chloroquine withdrawal; the parasites were exposed to the artesunate-mefloquine combination for about 10 years. Molecular analysis of antimalarial markers would aid the surveillance of antimalarial drug resistance in different areas with antimalarial drug resistance backgrounds and treatment policies.

## Materials and methods

### Sample collection and DNA extraction

**Fig. 1:**
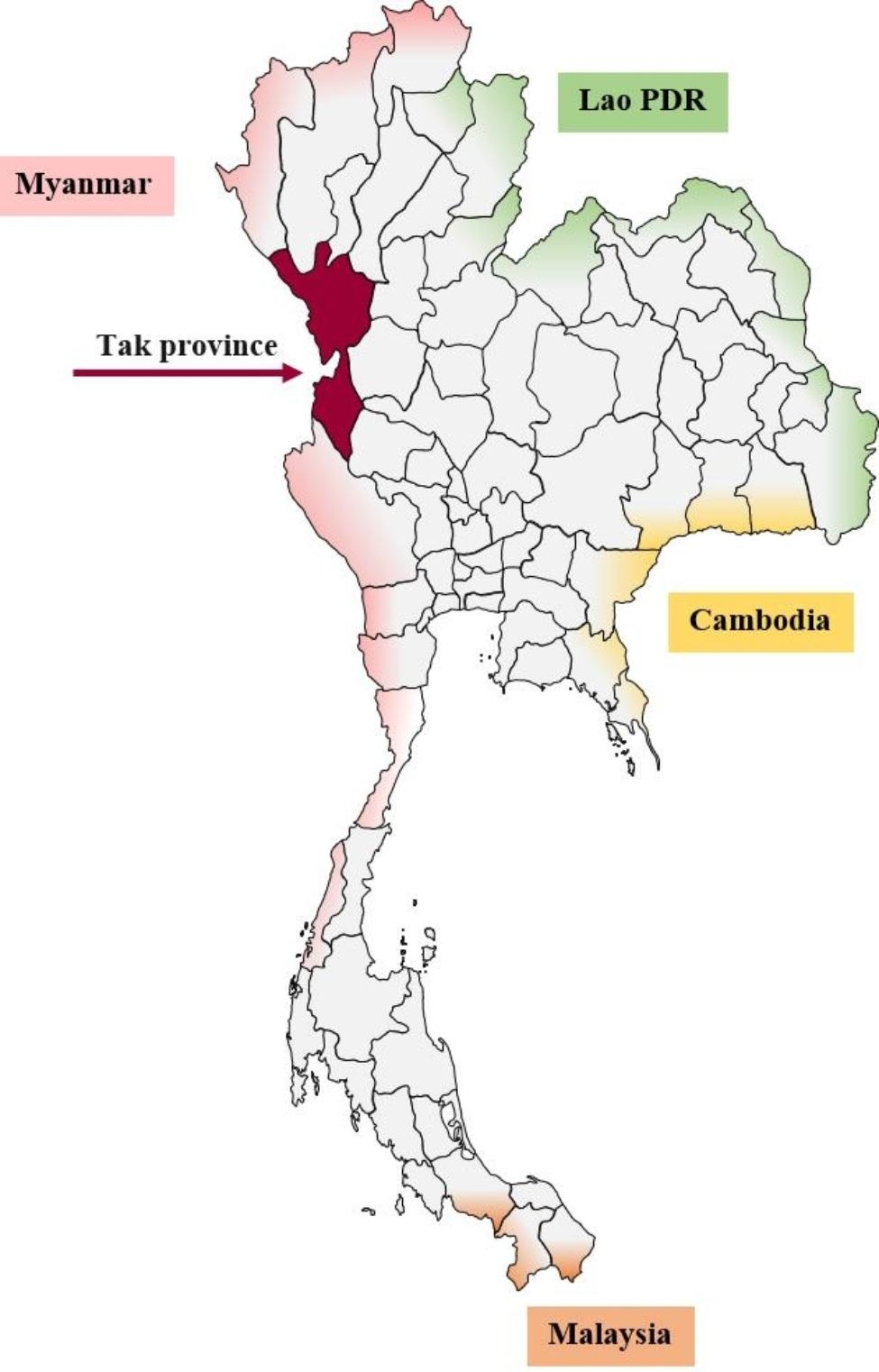
Map of Thailand indicating location of Tak province (arrow)

Six-hundred and eight whole blood or dried blood spot samples were collected from Thai, Burmese and Karen patients with confirmed *P. falciparum* infections who resided in Tak province (Mae Sot district), the malaria-endemic area along the Thai-Myanmar border (Fig. 1), during period 1 (1993–1997, 153 samples) and period 2 (2002–2008, 455 samples). The study protocol was approved by the Ethics Committee for Research in Human Subjects, Ministry of Public Health of Thailand (approval number 3/52–293) and the Human Research Ethics Commitee of Thammasat University-Medicine (approval number 068/2560). Written informed consents were obtained from all patients for participation in the main study to evaluate clinical efficacy and pharmacokinetics of the antimalarial regimens. The samples used in the analysis were leftover samples used in the primary studies as frozen whole blood and dried blood spot samples. The genomic DNA of each parasite isolate was extracted from the samples using DNA extraction commercial kits (QIAGEN, Hilden, Germany).

### Detection of ***pfcrt*** and ***pfmdr1*** polymorphisms by PCR-RFLP

Polymerase chain reaction-restriction fragment length polymorphism (PCR-RFLP) was used to detect *pfcrt* mutation at codons 76, 220, 271, 326, 356 and 371 [7, 8] and *pfmdr1* mutation at codon 86 [7]. PCR was performed in a total volume of 25 µl with the following reaction mixture: 0.4 µM of each primer, 2.5 mM MgCl_2_, 0.4 mM deoxynucleotides (dNTPs), 1x PCR buffer (100 mM KCl, 20 mM Tris-HCl pH 8.0), 0.2 unit of *Taq* DNA polymerase (Fermentas, Lithuania), and 2 µl of genomic DNA. The *P. falciparum* laboratory clones G112 (chloroquine-sensitive) and K1 (chloroquine-resistance) served as control.

### Determination of ***pfmdr 1*** copy number by real-time PCR

*Pfmdr1* gene copy number was determined by SYBR Green I real-time PCR [17] using *pfmdr1* sense 5’-GTGAGTTCAGGAATTGGTAC-3’, *pfmdr1* antisense (5’-GCCTCTTCTAT AATGGACATGG-3’), β-actin sense (5’-CCAGCTATGTATGTTGCT ATTC-3’), and β-actin antisense (5’-CTCCACTATCTAGGAATTGGTAC-3’) primers. The individual real-time PCR reaction was carried out in a 25 µl reaction volume containing 0.5x of iTaq™ Universal Supermixes (Bio-rad, USA), 1 µM each of sense and antisense primer, and 2 µl of genomic DNA. DNA was amplified using Bio-Rad CFX96 Real-Time PCR System (Bio-rad, USA). Cycle threshold (Ct) and melting curves were generated and used for data analysis. The copy number of *pfmdr1* was determined using the comparative C_t_ method (2^–ΔΔCt^ method) [17]. The 3D7 and Dd2 *P. falciparum* clones carrying 1 and 4 copies of *pfmdr1* were used as the reference controls. For each experiment, ΔCt_E_ denotes the ΔCt of each sample normalized to the reference gene (*pfβ-actin*) and was calculated as C_t,*target*_ - C_t,*pfβ−actin*_. ΔΔC_tE_ that normalized ΔCt_E_ to the ΔC_t_ of 3D7 was calculated (ΔΔC_tE_ = ΔC_tE_ - ΔC_t,3D7_). The *pfmdr1* copy number of each sample was calculated according to 2^–ΔΔCt,E^.

### Detection of ***pfk13*** mutations

For the detection of mutation on the *Kelch13* propeller domain of *P. falciparum*, nested PCR was performed to amplify 849 bp PCR product (at codon 427–709) [14]. The primers used included: first PCR (K13_PCR_F: 5’-CGG AGTGACCAAATCTGGGA-3’ and K13_PCR_R: 5’-GGGAATCTGGTGGTAACAGC-3’) and nested PCR (K13_N1_F: 5’-GCCAAGCTGC CATTCATTTG-3’ and K13_N1_R: 5’-GCC TTGTTGAAAGAAGCAGA-3’). The first PCR amplification was performed in a total volume of 20 µl with the following reaction mixture: 0.25 µM of each primer, 2.5 mM MgCl_2_, 0.2 µM deoxynucleotides (dNTPs), 1x PCR buffer (100 mM KCl, 20 mM Tris-HCl pH 8.0), 1.25 unit *Taq* DNA polymerase (Fermentas, Lithuania), and 5 µl of sample and control (3D7) genomic DNA. After an initial denaturation step at 95 °C for 15 min, the DNA template was subjected to 30 cycles of amplification: 30 s at 95 °C, 2 min at 58 °C, and 2 min at 72 °C, followed by a final extension at 72 °C for 10 min. The secondary PCR reaction was performed in a total volume of 45 µl with the following reaction mixture: 0.25 µM of each primer, 2.5 mM MgCl_2_, 0.2 µM deoxynucleotides (dNTPs), 1x PCR buffer (100 mM KCl, 20 mM Tris-HCl pH 8.0) ), 2.0 unit of *Taq* DNA polymerase (Fermentas, Lithuania), and 5 µl of PCR product from the first amplification. After pre-denaturation at 95 °C for 15 min, the reaction was amplified through 40 cycles: 30 s at 95 °C, 1 min at 60 °C, and 1 min at 72 °C, followed by a final extension at 72 °C for 10 min. Each PCR product (40 µl) was subsequently sent to the outsourced company (U2Bio, Bangkok) for sequencing. The consensus of forward and reverse sequences were generated using the Bioedit program and aligned with the K13 sequence of the 3D7 strain (PF3D7_1343700) as the reference strain using Basic Local Alignment Search Tool (BLAST) [18].

### Statistical analysis

Categorical variables, such as proportions of gene polymorphisms or gene copy numbers, are summarized as numbers and percentages. The differences in these variables between the two sample collection periods were analyzed using non-parametric tests, specifically the Chi-square test, with a statistical significance level set at α = 0.05 (SPSS version 15; SPSS, Chicago, Illinois,USA).

## Results

**Fig. 2:**
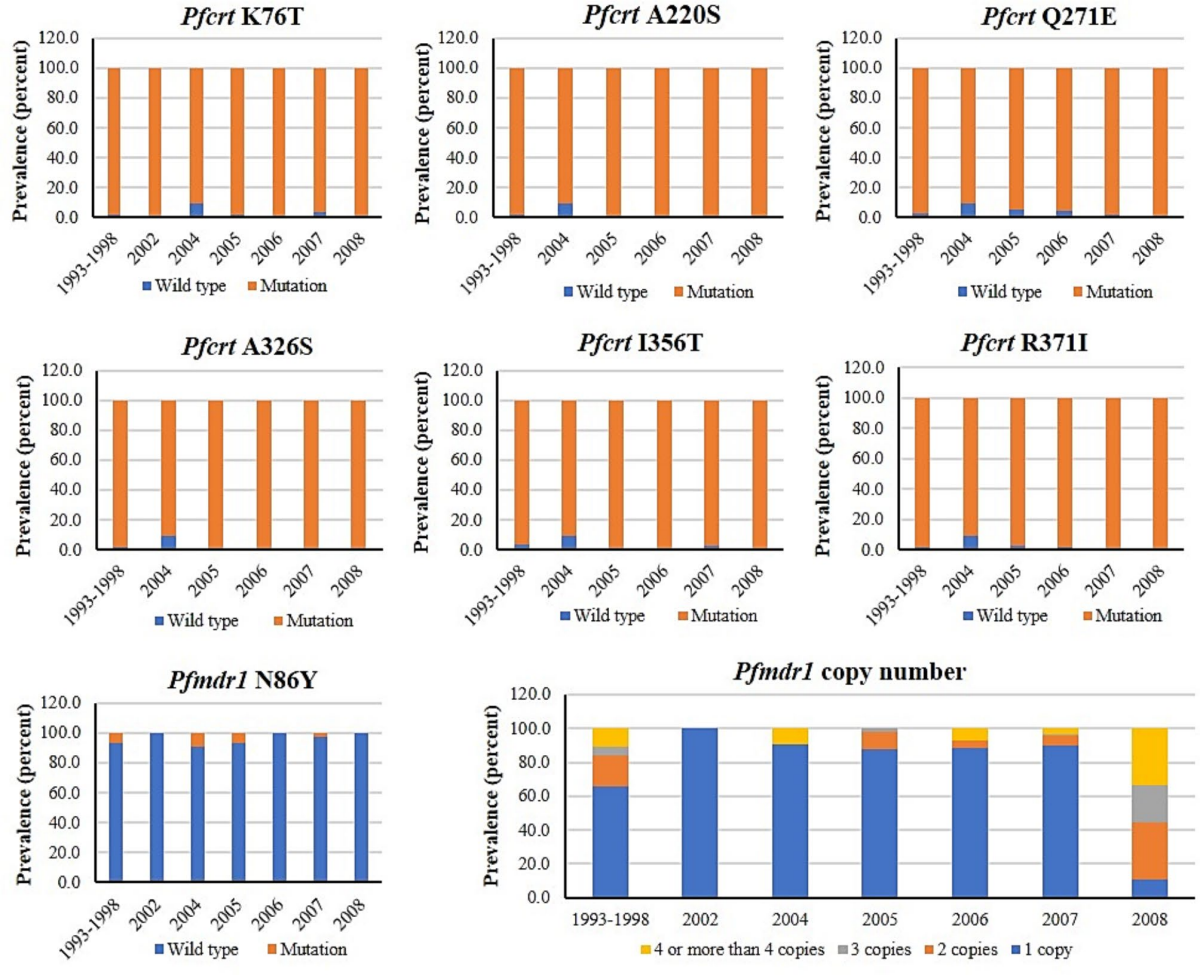
Distribution patterns of *pfcrt* and *pfmdr1* polymorphisms in *P. falciparum* isolates collected during period 1 (1993–1998) and period 2 (2002–2008). Significant differences in the prevalence of *pfcrt* A326S (*p* = 0.003) and *pfmdr1* copy number (*p* < 0.0001) were found during years of sample collection

The patterns and distribution of the three genes associated with antimalarial resistance in *P. falciparum* during period 1 (1993–1998) and period 2 (2002–2008) are summarized in Fig. 2; Table 1.

**Table 1:** Distribution patterns of *pfcrt* and *pfmdr1* polymorphisms in *P. falciparum* isolates collected during period 1 (1993–1998) and period 2 (2002–2008). Data are presented as percent and (number)

Gene	Codon	Genotype	Prevalence (*n*)		*p*-value
			Period 1	Period 2	
*Pfcrt*	76	Mutant	97.9% (137)	97.1% (401)	0.448
		Wild-type	2.1% (3)	2.9% (12)	
	220	Mutant	97.9% (140)	99.4% (171)	0.245
		Wild-type	2.1% (3)	1.0% (2)	
	271	Mutant	97.2% (139)	97.1% (333)	0.943
		Wild-type	2.8% (4)	2.9% (10)	
	326	Mutant	98.6% (140)	99.7% (385)	0.119
		Wild-type	1.4% (2)	0.3% (1)	
	356	Mutant	96.4% (134)	98.2% (378)	0.229
		Wild-type	3.6% (5)	1.8% (7)	
	371	Mutant	97.8% (136)	98.9% (375)	0.335
		Wild-type	2.2% (3)	1.1% (4)	
*Pfmdr1*	86	Mutant	7.1% (10)	3.1% (12)	0.038*
		Wild-type	92.9% (130)	96.9% (379)	
	Copy number	1 copy	65.7% (90)	87.8% (390)	< 0.0001*
		2 copies	18.2% (25)	6.3% (28)	
		3 copies	5.1% (7)	1.4% (6)	
		≥ 4 copies	10.9% (15)	4.5% (20)	

* Significant differences in polymorphisms proportion between the two sample collecting periods (*p* < 0.05)

### Pfcrt polymorphisms

The prevalence of *pfcrt* mutations at all codons (76, 220, 271, 326, 356, and 371) were found in almost all samples (96.4–99.7%) with no difference during the two periods. The proportions of mutation genotypes at codons 76, 220, 271, 326, 356 and 371 in the isolates collected during 1993–1998 compared with 2002–2008 were 97.9 vs. 97.1%, 97.9 vs. 99.4%, 97.2 vs. 97.1%, 98.6 vs. 99.7%, 96.4 vs. 98.2% and 97.8 vs. 98.9%, respectively.

### Pfmdr1 polymorphisms and amplification

Almost all isolates carried the wild-type genotype of *pfmdr1* gene at codon 86. The prevalence of N86 wild-type was significantly different between the two collecting periods (period 1 vs. period 2: 92.9 vs. 96.9%, *p* = 0.038). The patterns of *pfmdr1* copy number in samples collected during periods 1 and 2 were significantly different (*p* < 0.0001). Most carried 1 gene copy (65.7 vs. 87.8%), followed by 2 copies (18.2 vs. 6.3%), ≥ 4 copies (10.9 vs. 4.5%), and 3 copies (5.1 vs. 1.4%).

### Pfk13 polymorphisms

Investigation of *pfk13* polymorphism was successful in 68 of 178 selected samples (37, 2, 4 and 25 samples for 1993–1998, 2005, 2006 and 2007, respectively). No mutation of *pfk13* was found in any sample.

## Discussion

Drug resistance monitoring commonly involves *in vivo* studies to evaluate antimalarial efficacy and in vitro studies to determine the susceptibility of the parasite isolates to antimalarial drugs. Analysis of reliable molecular markers of antimalarial drug resistance provides advantages over *in vivo* and in vitro studies concerning the simplicity of methodology, low cost, and high throughput analysis from simple finger-prick blood spot samples [19]. The present study investigated the distribution patterns of the three valid molecular markers of antimalarial drug resistance *pfcrt*, *pfmdr1* and *pfk13* in 608 *P. falciparum* isolates collected during the two periods, 1993–1998 (period 1) and 2002–2008 (period 2) in malaria-endemic areas along the Thai-Myanmar border. Period 1 represents *P. falciparum* population in Thailand approximately 30 years after chloroquine withdrawal and the beginning of the clinical use of a 2-day artesunate-mefloquine combination. Period 2 represents *P. falciparum* population about 40 years after chloroquine withdrawal and ten years after the beginning of a 2-day artesunate-mefloquine combination, and the introduction of a 3-day artesunate-mefloquine combination (2008) [20].

*Pfcrt* polymorphism is the valid marker of sensitivity of *P. falciparum* to chloroquine. The in vitro sensitivity investigation showed that chloroquine-resistant isolates carried mutations at codons 76, 220, 271, 326, 356 and 371, while the sensitive isolates carried wild-type genotypes [21]. Besides chloroquine, *pfcrt* mutation at codon 356 has been associated with reducing the sensitivity of the parasite to quinine and pyronaridine but increasing sensitivity to mefloquine [22]. Despite the fact that chloroquine has been withdrawn from clinical use for the treatment of *P. falciparum* malaria in Thailand for several decades, the proportions of *pfcrt* mutations at the amino acid codons 76, 220, 271, 326, 356 and 371 remained remarkedly high (96.4–99.7%) in isolates collected during the two periods. The distribution pattern of 76T *pfcrt* mutation found in the current study was similar to that was reported in *P. falciparum* isolates collected from the same area in 2009 [12]. Surveillance of *pfcrt* mutation was also conducted in several malaria-endemic countries for the possibility of the re-introduction of chloroquine for *P. falciparum.* The 76T *pfcrt* mutation persisted in Nigeria after 11 years of chloroquine withdrawal [23]. Similarly, a markedly high prevalence (> 90%) of the 76T mutation was observed in Uganda, Gabon, Tanzania, Iran, Liberia, and Benin [24]. On the other hand, the prevalence has constantly dropped after chloroquine withdrawal in Zambia from 2002 to 2016 [25], Mali from 2006 to 2010 [26], Malawi from 1993 to 2001 [27, 28], Senegal from 2000 to 2005 [29], Kenya from 1993 to 2006 [30] and China from 1978 to 2001 [31]. Differences in *pfcrt* mutation distribution patterns could be due to differences in disease epidemiology, transmission intensity, host immunity, mutation compensatory, infection clonality, and treatment policy in different countries [32]. Surveillance of *pfcrt* conducted during 1996–2009 in the Karen state of Myanmar, located nearby the area of Mae Sot district of Thailand, revealed the impact of chloroquine pressure and population migration on *pfcrt* 76T mutation; almost all isolates (95–100%) were chloroquine resistance [33]. In addition, the studies conducted in the same area during 2009–2010 also revealed the prevalence of *pfcrt* mutation of higher than 90% [12, 34]. This may suggest the impact of chloroquine pressure and population migration on chloroquine resistance in these areas. It is noted for the absence of reversal of sensitivity of *P. falciparum* to chloroquine in Thailand since chloroquine has continuously been used as the first-line treatment for *P. vivax* and other non-*P. falciparum* malaria [6]. Thailand is a country with low malaria transmission, and patients usually have low or lack of acquired immunity to malaria [35]. Treatment with antimalarial drugs is recommended in all cases with clinical signs and symptoms of malaria. This results in subtherapeutic drug concentrations in blood and the risk of selective pressure and drug resistance [36]. In such low transmission areas as Thailand, drug-resistant genotypes can be maintained in parasite populations even after the termination of drug pressure [37]. Not only can chloroquine, but also other antimalarial drugs pressure maintain *pfcrt* mutant isolates. The emergence of *pfcrt* mutation was reported to be induced by stepwise selection with amantadine or halofantrine [38]. Increasing the number of nonsynonymous modifications in *pfcrt* throughout malaria-endemic areas in Thailand could occur through drug pressure from the increased duration of artesunate-mefloquine combination regimen administration to 3 days or the accumulative effect of mefloquine usage [20].

The *pfmdr1* polymorphisms, both point mutation and gene amplification, affect the sensitivity of *P. falciparum* to several antimalarial drugs. Five resistance-associated SNPs (single nucleotide polymorphisms) of *pfmdr1* have been identified, of which the N86Y and Y184F are more common in Asia and Africa, while S1034C, N1042D, and D1246Y are found more frequently in South America [39]. *Pfmdr1* mutation at codon 86 (86Y) augments *P. falciparum* resistance to chloroquine and the ACT partner drug amodiaquine but increases parasite susceptibility to lumefantrine, mefloquine, and dihydroartemisinin [40]. The initial clinical usage of mefloquine as an MSP combination in Thailand since 1985 may initiate mefloquine pressure and result in the development and spread of the mefloquine-resistant parasite. Even though the wild-type *pfmdr1* N86 was reported to select mefloquine resistance, the drug was still selected as the partner drug in the ACT [41]. A markedly high prevalence of *pfmdr1* N86 of 92.9% was found in isolates collected during period 1 (1993–1998). Thereafter, the intensive use of mefloquine as an artesunate-mefloquine combination in all falciparum malaria cases resulted in a significant increase in *pfmdr1* N86 prevalence up to 96.9% in samples collected during period 2 (2002–2008). Furthermore, the studies conducted in the same area during 2009–2010 also revealed *pfmdr1* point 86 (wild type) in all samples [12, 34].

Amplification of *pfmdr1* has been linked with decreased sensitivity of *P. falciparum* isolates to mefloquine and related compounds, including artemisinins [42, 43]. The observation of a high prevalence of isolates carrying more than one *pfmdr1* copy in samples collected since 1993 confirmed the spread of mefloquine-resistant *P. falciparum* in areas along the Thai-Myanmar border before the introduction of artesunate-mefloquine therapy. *Pfmdr1* amplification emerges at a higher rate than point mutation through stimulation by several antimalarial drugs with the linkage between gene dosage and drug resistance [44]. In the present study, most isolates collected during both periods carried only a single *pfmdr1* copy (period 1 vs. period 2: 65.7% vs. 87.8%). This may reduce the intensity of mefloquine resistance due to the large proportion of parasites carrying a single *pfmdr1* copy. For chloroquine, *pfmdr1* amplification is negatively associated with chloroquine sensitivity which can influence the de-amplification of *pfmdr1* to a single copy [45]. The decrease in the prevalence of *pfmdr1* amplification could lead to the selection of chloroquine-resistant *P. falciparum* strains. It was noted however that the large proportion of parasites carrying a single *pfmdr1* copy found in the study and inconsistent findings among studies in the same areas could reflect the actual situation, as well as study limitations of small sample size and quality of the samples, and differences in study sites and populations. A high prevalence of the isolates carrying *pfmdr1* gene amplification (88.9%, n = 8) was found in samples collected in 2008 (n = 9) (Fig. 2). The study conducted in Mae Sot district, Tak province in 2004 showed that 58% of the parasite isolates carried a single *pfmdr1* copy number [46]. An Increase in isolates’ prevalence harboring *pfmdr1* amplification to 53.7% was reported in a study conducted in the same area during 2009–2010 [34]. In addition, the study conducted during 1995–2007 in Karen population living along the Thai-Myanmar border found a gradual decrease in the proportion of the isolates carrying a single *pfmdr1* copy number as 70% (1996), 65% (2001), 49% (2005) and 47% (2006) [47]. The decreasing trend of the isolates carrying a single *pfmdr1* copy number was also found in the study conducted during 2003–2013, i.e., 50.8% (2005), 57.8% (2008), 26.1% (2011), and 35.3% (2013) [48]. Amplification of *pfmdr1* was reported to modulate the efficacy of the artesunate-mefloquine combination and the potential of malaria recrudescence [12, 48]. The recrudescence following this combination may be due to the high intensity of mefloquine resistance background and the emergence of artesunate-resistant parasites. Both could increase treatment failure rates due to selective pressure from sub-therapeutic drug levels [49].

Artemisinin resistance was first emerged in Cambodia in 2010 [50]. The *pfk13* mutation is currently proposed as a valid molecular marker for large-scale artemisinin resistance surveillance [51]. Correlation studies suggest common mechanisms of action and resistance of *P. falciparum* to lumefantrine, mefloquine, and halofantrine. *Pfk13* and *pfmdr1* and *pfcrt* polymorphisms may affect the access and/or action of these arylaminoalcohol drugs for hemoglobin digestion in the food vacuoles. Selective pressure from the use of lumefantrine or mefloquine in ACTs may drive the selection of *pfk13* polymorphisms along with the mutations of *pfmdr1* and *pfcrt* associated with lower susceptibility to these drugs [52]. The *pfk13* polymorphisms that have previously been reported include F446I, N458Y, R561H, P574L (commonly found in western Thailand, Myanmar, and China); C580Y (commonly found in Cambodia, Vietnam, and Laos); P553L (commonly found in western Thailand, Myanmar, China, Cambodia, Vietnam, and Laos); and A578S (commonly found in Africa) [53]. Furthermore, the following polymorphisms, i.e., I437T, V445G, F451I, E455K, D464E, A481T, A504T, D512N, R513H, R529K, T535A, E567D, T573A, M608V, L618L (TTa 1857 TTg) and V637A have been reported in *P. falciparum* isolates from the Thai-Myanmar border during 2006–2010 [16]. The present study investigated the prevalence of *pfk13* polymorphisms in the samples collected from the Thai-Myanmar border during 1993–1998 and 2002–2008, which were the period of artesunate-mefloquine introduction as the first-line treatment of falciparum malaria. Although several SNPs related to artemisinin resistance have been reported, none of the mutations has been observed along the K13 propeller domain protein position 427–709. Limitations of the study include small sample size; particularly those collected during 1993–1998, quality of the samples, as well as differences in study sites and populations. These factors could have affected the results of *pfk13* polymorphisms reported in this study. Retrospective investigation is important to determine the prevalence of *pfk13* polymorphisms during 1993–1998 and 2002–2008; nevertheless, the availability of previously collected samples did not reach the number obtained from sample size estimation.

Long-time sample storage, type of samples, and parasitemia were the main factors affecting the low success rate of K13 propeller domain protein mutation analysis (38.2%, 68 of 178 samples). In addition, the small sample size reduced the chance to observe any polymorphism if it existed. Nevertheless, the study conducted in the same area in 2004 also showed 100% of the isolates carrying wild-type K13 propeller domain protein [46]. In another study conducted during 2003–2010, the mutation was observed at a prevalence of 17.4% [16]. The study conducted in the Karen population during 2003–2013 found increased K13 mutation in any SNPs, i.e., 6.7% (2003), 2.0% (2004), 14.9% (2005), 26.7% (2008), and 83.9% (2013) [47]. A clinical study conducted during 2008–2009 revealed the decline in the clinical efficacy of artesunate-mefloquine combination with 42- and 28-day cure rates of 72.58% and 83.06%, respectively [55]. Dihydroartemisinic-piperaquine combination therapy later replaced this combination in 2015 [15].

## Conclusions

The distribution of antimalarial drug resistance molecular markers effectively reflects the history of antimalarial drug usage, which is crucial for supporting malaria control efforts. In Thailand, the persistent presence of *pfcrt* mutations in *P. falciparum* isolates can be attributed to chloroquine selective pressure resulting from continued drug use for non-*P. falciparum* malaria treatment. Therefore, the prophylactic use of chloroquine to prevent malaria infection is not recommended. Additionally, the use of ACT regimens containing mefloquine should be avoided due to the high prevalence of mefloquine-resistant *P. falciparum* isolates. The emergence and spread of *P. falciparum* resistant to the dihydroartemisinin-piperaquine combination is concerning, especially in the context of multi-drug-resistant parasites. The validation of molecular markers related to *P. falciparum* antimalarial drug resistance is essential to support surveillance efforts to assess the clinical efficacy of this combination regimen.

## Data Availability

All data and materials used in this manuscript will be available upon request. For further information, please contact the corresponding author, Kesara Na-Bangchang, *via* email at kesaratmu@yahoo.com.
